# Care of HIV-Infected Pregnant Women in Maternal–Fetal Medicine Programs

**DOI:** 10.1155/S1064744901000151

**Published:** 2001

**Authors:** Peter A. Sklar, Susanne L. Bathgate, Heather A. Young, David M. Parenti

**Affiliations:** 1 Division of Infectious Diseases Department of Medicine The George Washington University Medical Center 2150 Pennsylvania Avenue, N.W., Suite 5-411 Washington, DC 20037 USA; 2 Division of Maternal-Fetal Medicine Department of Obstetrics and Gynecology The George Washington University School of Medicine and Health Sciences Washington, DC USA; 3 Department of Biostatistics The George Washington University School of Public Health and Health Services Washington, DC USA

## Abstract

*Objective:* To survey the evolution over the past decade of attitudes and practices of obstetricians in maternal–fetal medicine fellowship programs regarding the management of human immunodeficiency virus (HIV)-infected pregnant women.

*Methods:* Directors of all 65 approved maternal–fetal medicine training programs were sent questionnaires, responses to which were to reflect the consensus among members of their faculties. Programs were stratified based upon the number of HIV-infected pregnant patients cared for in the previous year.

*Results:* Responses reflect experience with over 1000 infected pregnantwomen per year, nearly one-quarter with advanced disease. Combination antiretroviral therapy was prescribed by all respondents, universally in the 2nd and 3rd trimesters. A three-drug regimen (often containing a protease inhibitor) was used more often by those who treated at least 20 HIV-infected pregnant patients per year than by those programs seeing a lower number of patients (80 vs 59%).Despite the known and unknown risks of the use of antiretrovirals during pregnancy, only half of all responding programs report adverse events to the Antiretroviral Pregnancy Registry; reporting was more common among the institutions seeing a higher number of patients (61 vs 45%). Seventy-eight percent of higher volume programs enroll their patients in clinical studies, usually multicenter, versus 35% of lower volume programs.

*Conclusions:* Care for HIV² pregnant women has dramatically changed over the past decade. Antiretroviral therapy is now universally prescribed by physicians involved in maternal–fetal medicine training programs. Given limited experience with these agents in the setting of pregnancy, it is essential for maternal–fetal medicine practitioners to actively report on adverse events and participate in clinical trials.


Infect Dis Obstet Gynecol 2001;9:81–87

### Care of HIV-infected pregnant women in maternal–fetal medicine programs

**Peter A. Sklar[1], Susanne L. Bathgate[2], Heather A. Young[3] and David M. Parenti[1]**

[1]*Division of Infectious Diseases, Department of Medicine, and* [2]*Division of Maternal-Fetal Medicine, Department of Obstetrics and Gynecology, The George Washington University School of Medicine and Health Sciences, Washington, DC*
[3]*Department of Biostatistics, The George Washington University School of Public Health and Health Services, Washington, DC*

***Objective:*** To survey the evolution over the past decade of attitudes and practices of obstetricians in maternal–fetal medicine fellowship programs regarding the management of human immunodeficiency virus (HIV)-infected pregnant women.
***Methods:*** Directors of all 65 approved maternal–fetal medicine training programs were sent questionnaires, responses to which were to reflect the consensus among members of their faculties. Programs were stratified based upon the number of HIV-infected pregnant patients cared for in the previous year.
***Results:*** Responses reflect experience with over 1000 infected pregnant women per year, nearly one-quarter with advanced disease. Combination antiretroviral therapy was prescribed by all respondents, universally in the 2nd and 3rd trimesters. A three-drug regimen (often containing a protease inhibitor) was used more often by those who treated at least 20 HIV-infected pregnant patients per year than by those programs seeing a lower number of patients (80 *vs* 59%). Despite the known and unknown risks of the use of antiretrovirals during pregnancy, only half of all responding programs report adverse events to the Antiretroviral Pregnancy Registry; reporting was more common among the institutions seeing a higher number of patients (61 *vs* 45%). Seventy-eight percent of higher volume programs enroll their patients in clinical studies, usually multicenter, versus 35% of lower volume programs.
***Conclusions:*** Care for HIV+ pregnant women has dramatically changed over the past decade. Antiretroviral therapy is now universally prescribed by physicians involved in maternal–fetal medicine training programs. Given limited experience with these agents in the setting of pregnancy, it is essential for maternal–fetal medicine practitioners to actively report on adverse events and participate in clinical trials.

Key words: HIV, VERTICAL DISEASE TRANSMISSION, ANTI-HIV AGENTS

Vertical transmission of human immunodeficiency virus (HIV) has been dramatically reduced in the United States and other countries where anti-retroviral therapy is widely available, where alternatives to breastfeeding are a viable option, and where elective Cesarean deliveries are selectively performed[1]. The relevance of mother-to-child transmission in the United States stems from the fact that new HIV infections are most predominant among young women of

Correspondence to: Peter Sklar, MD, MPH, Division of Infectious Diseases, The George Washington University Medical Center, 2150 Pennsylvania Avenue, N.W., Suite 5-411, Washington, DC 20037. E-mail: peter.sklar@alumni.duke.edu reproductive age, particularly adolescent minorities[2]. Data on interventions to reduce the rate of transmission have rapidly evolved over the past decade. A placebo-controlled trial demonstrating clear benefits of the administration of antiviral therapy to pregnant women and neonates was published in 1994: the Pediatric AIDS Clinical Trials Group (PACTG) 076 study[3]. The impact of this regimen has been proven in follow-up epidemiologic studies, demonstrating a reduction in perinatal AIDS cases in the United States from the years 1992 to 1997 by 67%[4]. A significant proportion of this benefit is thought to be related to the reduction of plasma HIV-1 viral load as demonstrated in the PACTG 185 study and the Women and Infants Transmission Study (WITS), enrolling women with more advanced disease than those in the PACTG 076 group[5,6]. Scheduled Cesarean delivery, particularly for mothers with high plasma levels of HIV, performed before the onset of labor and before the rupture of membranes, has proven to be effective in reducing the rate of transmission of virus as evidenced in a meta-analysis performed by the International Perinatal HIV Group. However, most of the studies in that analysis were conducted before the widespread use of highly active antiretroviral therapy (HAART)[7].

The dissemination of information on the management and therapy of HIV-infected pregnant women has come via both public and private sources. The US Department of Health and Human Services Public Health Service publishes recommendations for the use of antiretroviral therapy in HIV-infected pregnant women. The most recent revision was in February 2001; at the time this survey was performed, the most recent revision had been on January 30, 1998. The current guidelines strongly recommend the use of these agents for the purposes of addressing two separate but related issues – antiretroviral therapy to treat the mother's HIV infection and antiretroviral chemoprophylaxis to reduce the risk of perinatal HIV-1 transmission[8]. The American College of Obstetricians and Gynecologists (ACOG) has issued several policy statements and committee opinions regarding the treatment of HIV-infected pregnant women, which are constantly being updated and revised[9–12].

For more than 26 years the American Board of Obstetrics and Gynecology (ABOG) has certified obstetrician–gynecologists for special competence in maternal–fetal medicine. The 65 currently approved fellowship programs that train candidates for subspecialty certification are periodically reviewed for accreditation based upon demonstrated continued academic and clinical competence in the management of all complications of pregnancy. In 1991 an initial survey of the management and therapy of HIV-infected pregnancies in maternal–fetal medicine training programs was conducted[13]. Much has evolved in the field, leading to dramatic reductions in transmission, yet there is still a lack of uniformity in treatment strategies. The purpose of this study is to characterize current opinions and practices among maternal–fetal medicine practitioners and contrast them with the study performed in 1991.

### SUBJECTS AND METHODS

In August 1999 a 30-item questionnaire concerning issues in the management of HIV-infected pregnant women was sent to the directors of the 65 approved training programs in maternal–fetal medicine in the United States. This was in follow-up to an initial study conducted during 1991–1992. The directors were asked to answer each question in a manner reflecting the consensus among the members of their division. Two follow-up mailings and telephone contacts were made to facilitate questionnaire completion. By May 2000 the directors of 41 of the 65 programs (63%) had returned completed questionnaires and the data were compiled and analyzed.

Respondents were stratified into two groups based on the number of HIV-infected pregnant patients seen at their institution within the previous year – either greater than or equal to 20 patients (18 programs) or less than 20 patients (23 programs). Differences in responses were analyzed using either $\chi^2$ analysis or Fisher's exact test. Clinically significant trends were reported; they were not statistically significant unless otherwise noted. The SAS 6.12 statistical package was used in these analyses (SAS Institute, Inc., Cary, NC).

### RESULTS

Forty-one of the 65 currently approved fellowship training programs responded to our survey, representing a nationwide sample of programs with varied size among 23 states. There were 13 fewer active fellowship programs in 1999 than there were in 1991. The 1991 survey was based on the experience of 381 370 pregnancies – in 2105 of which the mother was HIV-positive (HIV+) – while the current survey is based on a sample of 143 037, in which 1007 of the mothers were HIV+. The data represent approximately one-sixth of the estimated 6000 HIV-infected women who deliver each year in the United States. Some large programs in New York, California and Illinois did not respond, for unknown reasons, and thus their experience is not reflected in the survey results. The programs which see 20 or more HIV+ pregnancies per year are actually seeing over three-quarters of all HIV+ pregnant women in this study. Five (12%) of the responding programs see fewer than five pregnancies complicated by HIV per year. The proportion of women with advanced (CD4+ cell count < 200 cells/mm$^3$) or symptomatic disease has increased from approximately 20% in 1991 to approximately 25% in 1999, despite the more widespread use of potent antiretroviral therapy. The clinics of maternal–fetal medicine fellowship training programs are more likely to register patients with HIV-related complications, thus women with advanced HIV disease may be overrepresented in our sample.

Table 1 presents the demographic characteristics of all respondents and of the stratified groups. There are 153 037 deliveries performed annually at the 41 maternal–fetal medicine programs surveyed (mean 3732.61; range 300–14 700 per institution). On average, approximately 1007 HIV-infected pregnant women (mean 24.56; range 0–130 per institution) receive care in the clinics of these training programs each year; 229 with CD4+ lymphocyte counts < 200 cells/mm$^3$ (mean 5.59; range 0–25 per institution). Thus, nearly one-quarter of all pregnancies complicated by HIV which were included in this survey involve women with advanced disease. Fifty-nine percent (24 of 41) of these programs have a special clinic for HIV-infected pregnant women. Eighty percent (33 of 41) of the program directors surveyed stated that additional medical specialists outside their divisional staff assist in the management of HIV-infected patients.

HIV testing is uniformly offered by all the responding programs; however, only 79% of patients consented to testing. Prenatal testing identified 406 new HIV diagnoses per year among the 41 surveyed institutions (mean 9.90; range 0–40). Thus 40% of all HIV-infected pregnant women in these institutions are being diagnosed as part of these prenatal screening efforts. Women presented for prenatal care, on average, during either the 1st (45%) or 2nd (55%) trimester.

Table 2 summarizes the responses of program directors regarding attitudes towards the use of antiretrovirals during pregnancy. Antiretrovirals were prescribed during pregnancy by all respondents, universally in the 2nd and 3rd trimesters. Although there are differences of opinion on whether or not to start antiretrovirals during the 1st trimester, none of the respondents would recommend stopping therapy if a patient was already receiving it. All surveyed program directors recommended the use of combination therapy (two or more drugs) during pregnancy. A three-drug regimen was used more often by those

**Table 1** Characteristics of responding institutions (totals (means))

| Characteristic | All respondents (n = 41) | Centers with: ≥ 20 HIV+ pregnancies per year (n = 18) | Centers with: < 20 HIV+ pregnancies per year (n = 23) |
|---|---|---|---|
| Deliveries/year | 153 037 (3732.61) | 85 677 (4759.83) | 67 360 (2928.70) |
| HIV+ deliveries / year | 1006.5 (24.55) | 783.5 (43.53) | 223 (9.70) |
| HIV+ deliveries / year with CD4 count < 200 cells/mm$^3$ | 228.5 (5.57) | 164 (9.11) | 64.5 (2.80) |

HIV+, human immunodeficiency virus-positive

**Table 2** Respondents' attitudes towards the use of antiretrovirals in pregnancy (*n* (%))

| *Characteristic* | *Centers with* ≥ 20 HIV+ pregnancies per year | *Centers with* < 20 HIV+ pregnancies per year |
|---|---|---|
| Prescription of antiretrovirals in first trimester | 17/18 (94.44) | 16/20 (80.00) |
| Selection of antiretrovirals based on prior exposure | 14/18 (77.78) | 13/21 (61.90) |
| Zidovudine (AZT) always part of regimen | 11/18 (61.11) | 17/23 (73.91) |
| Reporting of adverse events | 11/18 (61.11) | 10/22 (45.45) |
| Increased gestational diabetes on PIs | 3/18 (16.67) | 1/22 (4.55) |
| Altered screening practices for gestational diabetes in patients on PIs | 5/18 (27.78) | 3/22 (13.64) |
| PACTG 076 regimen – zidovudine dosing | | |
| Strict | 7/18 (38.89) | 12/22 (54.55) |
| Modified | 11/18 (61.11) | 10/22 (45.45) |
| Number of drugs included in antiretroviral regimen | | |
| Two drugs | 3/15 (20.00) | 7/17 (41.18) |
| Three drugs | 12/15 (80.00) | 10/17 (58.82) |

HIV, human immunodeficiency virus; PI, protesae inhibitor; PACTG, Pediatric AIDS Clinical Trials Group who treat more than 20 HIV+ patients per year (80 *vs* 59%). The most common modification of the PACTG 076 regimen, which is based on zidovudine monotherapy, involved a change in the oral zidovudine dosing to twice-a-day dosing, with the addition of lamivudine (Combivir® (GlaxoSmithKline, Research Triangle Park, NC)). Other modifications involved the co-administration of a protease inhibitor as part of a three-drug regimen. Zidovudine still remained part of the prescribed regimen for the majority of respondents (68%). Strict inclusion of zidovudine as part of a combination regimen was more common in the group seeing fewer than 20 HIV+ pregnancies per year (74 *vs* 61%). Sixty-nine percent of all respondents took into account prior antiretroviral exposure when designing a treatment regimen, more commonly in the experienced group of providers (78 *vs* 62%). Eighty-seven percent of surveyed institutions routinely measured HIV-1 RNA levels, which are used to assist in treatment recommendations.

When asked if there were specific antiretroviral agents that were avoided during pregnancy, only 13 of 41 programs responded. Of those 13, 10 specifically mentioned efavirenz, most citing its teratogenic potential as demonstrated in animal models. Others listed some of the newer agents (abacavir, amprenavir), citing unfamiliarity or minimal experience with these agents. One program director cited indinavir, and mentioned concerns about drug-induced hyperbilirubinemia and its potential effects on the fetus. Despite the known and unknown risks of the use of antiretrovirals in pregnancy, only half of all programs were reporting adverse events to the Antiretroviral Pregnancy Registry (see below); reporting was more common in those who saw 20 or more HIV+ pregnancies per year (61 *vs* 45%). Respondents were surveyed as to whether they had noticed an increased incidence of gestational diabetes in their patients on protease inhibitors; the majority (90%) had not. However, several programs had altered their screening practices for gestational diabetes, a situation more common among those programs seeing more HIV-infected pregnant mothers (28 *vs* 14%). The overall strategy of these altered screening practices was to begin earlier in pregnancy and to screen more than once.

Program directors were asked about the practice patterns of their divisions in relation to the use of specific algorithms for managing HIV in pregnancy and regarding participation in clinical studies in the management of HIV infection in pregnancy. It was more common for programs seeing a larger number of patients to have developed such an algorithm for managing HIV in pregnancy (78 *vs* 35%). Seventy-eight percent of programs which saw 20 or more HIV+ pregnancies per year enrolled their patients in clinical studies, as compared with 35% of those which see less than 20; this difference was statistically significant ($p = 0.006$). Almost universally, these studies were multicenter.

Eighty percent of responding institutions offer termination of pregnancy to HIV-infected pregnant patients; acceptance was 9% on average. Sixty-three percent of institutions would recommend elective Cesarean sections to those HIV-infected pregnant patients not on HAART; if on HAART the recommendation would only be made by 35% of programs. Seventy-nine percent of responding program directors reported that the standard in their practices was to alter obstetric techniques in pregnancies complicated by HIV. The most common alterations were avoiding early rupture of membranes and artificial rupture of membranes, active management of labor to expedite delivery, avoiding the use of scalp electrodes and other forms of invasive monitoring, avoiding the use of forceps, and avoiding the performance of episiotomies. Over half (53%) of respondents perceived that pregnant women infected with HIV were at increased risk of maternal–fetal complications; this perception was strongest among the programs seeing the majority of HIV-complicated pregnancies (61 *vs* 45%). Specific questions were asked regarding recommendations for and performance of amniocentesis/chorionic villus sampling. The majority of institutions would offer amniocentesis or both if indicated and desired (32% amniocentesis alone, 32% both). There was almost unanimous recommendation against breastfeeding (95%). Interestingly, the two dissenting institutions were among the group seeing a higher volume of HIV-infected pregnant women.

### DISCUSSION

The past decade has been one of extraordinary progress in our understanding of the epidemic of vertically transmitted HIV. Through the efforts of ACOG, the Society for Maternal–Fetal Medicine, the US Public Health Service and others, this information has been disseminated, establishing updated standards of care with great success. The results of this study demonstrate, however, that there are still areas of considerable controversy. While the benefits of HAART administered to women to alter the progression of their own HIV disease are clear, whether the use of these regimens in pregnancy, particularly when the maternal HIV-1 RNA level can be suppressed below 1000 copies/ml, will provide superior risk reduction over the use of zidovudine or the performance of elective Cesarean section is not yet known.

Many of the changes in practice identified since 1991 are a reflection of advances in the care of all patients with HIV infection. The current survey was meant to pool experience and opinions relating to newer concepts in the field of maternal–fetal medicine; when appropriate, comparisons were made with the initial 1991 survey. Recall bias may have affected some of the estimates reported by the program directors. However, as part of the recertification process, fellowship program directors are required periodically to report the size and composition of their obstetric population to ABOG. It has become increasingly common for institutions to have a special clinic (i.e. separate from the general obstetrics clinic) set up to care for pregnant patients with HIV infection; in 1999 59% of responding programs had established such a clinic, while in 1991 only 40% had established one. The assistance of experts outside divisions of maternal–fetal medicine, such as infectious disease specialists, remains very common, although there has been a decline (85% of respondents in 1991, 80% in 1999). More institutions have developed specific algorithms for the management of these patients (56% in 1991, 63% in 1999).

The perception that HIV-infected pregnant women are at higher risk of obstetric complications has remained constant over the period 1991–1999 at approximately 50% of respondents. However, the trend towards offering/recommending termination of HIV+ pregnancies has decreased from 90% in 1991 down to 80% in 1999, perhaps reflecting increased confidence in the use of antiretroviral therapy and other measures to reduce vertical transmission. In regard to antiretroviral therapy, in 1991 the only option available was zidovudine. Although the initial survey was conducted prior to the publication of the results of the PACTG 076 study, there was still much anticipation of the potential benefits of antiviral therapy in reducing mother-to-child transmission, with 74% believing that zidovudine should be offered to asymptomatic HIV-infected pregnant women and 95% believing that it should be offered to symptomatic patients. In 1999 antiretrovirals were universally recommended.

The results of PACTG 367 give support to the results of this survey. This study represents a retrospective abstraction of prenatal data from one of the PACTG sites (464 women)[14]. Of HIV-infected women who delivered at that site between January 1998 and May 1999, 54% knew their HIV status prior to pregnancy while 44% were diagnosed during pregnancy, similar to the approximately 40% noted in our survey. Stage of disease, as approximated by CD4+ cell count, was also similar between our population and that in the PACTG, with 20% and 25%, respectively, having counts $< 200$ cells/mm$^3$.

Drugs such as protease inhibitors and non-nucleoside reverse transcriptase inhibitors (NNRTI) have only been available for the past 6 years; their long-term toxicities in adults, let alone their effects *in utero*, are still largely unknown. Some results from French and Swiss studies – not substantiated in the United States experience – give cause for concern, as they suggest potential mitochondrial toxicity from the use of nucleoside reverse transcriptase inhibitors (NRTI) and potential premature births from the use of protease inhibitors[15,16]. In the most recent Public Health Service guidelines, coadministration of stavudine (d4T) and didanosine (ddI) for HIV-infected women has been discouraged secondary to reports of maternal morbidity related to lactic acidosis[8]. Alarmingly, only 50% of responding programs submitted information on adverse effects to the Antiretroviral Pregnancy Registry. The purpose of this registry is to collect data on antiretroviral exposure during pregnancy to assess the teratogenic potential of these drugs. It is a collaborative project of the pharmaceutical manufacturers with an advisory committee of obstetric and pediatric practitioners. Given the limitations in our knowledge base, it is essential that data be collected on the experience of all pregnancies where antiretrovirals are used, not just at the higher volume centers (Antiretroviral Pregnancy Registry, 140 Commonwealth Drive, Wilmington, NC 28403; phone: (800) 258-4263, fax: (800) 800-1052).

It is also of utmost importance for studies to be conducted evaluating the impact of protease inhibitors on the development of gestational diabetes, a significant cause of morbidity for both mother and child. A growing body of evidence implicates these agents as causative agents of the lipodystrophy syndrome, characterized by insulin resistance, dyslipidemia and fat redistribution. Another area for future research relates to the implications of antiretroviral resistance developing in women and children exposed to these agents in the perinatal period.

### CONCLUSION

In many ways, vertically transmitted HIV is a very preventable disease; eradication is within the realm of possibility in countries with the resources of antiretrovirals and alternatives to breastfeeding.

### ACKNOWLEDGEMENT

The authors thank John H. Grossman, MD, PhD, for his guidance and support with this project.

### REFERENCES

1. Joint United Nations Programme on HIV/AIDS (UNAIDS). Report on the global HIV/AIDS epidemic. Geneva, Switzerland: UNAIDS, June 2000
2. Rosenberg PS, Biggar RJ. Trends in HIV incidence among young adults in the United States. *JAMA* 1998;279:1894–9
3. Connor EM, Sperling RS, Gelber R, *et al*. Reduction of maternal-infant transmission of human immunodeficiency virus type 1 with zidovudine treatment. Pediatric AIDS Clinical Trials Group Protocol 076 Study Group. *N Engl J Med* 1994;331:1173–80
4. Lindegren ML, Byers RH, Thomas P, *et al*. Trends in perinatal transmission of HIV/AIDS in the United States. *JAMA* 1999;282:531–8
5. Mofenson LM, Lambert JS, Stiehm ER, *et al*. Risk factors for perinatal transmission of human immunodeficiency virus type 1 in women treated with zidovudine. *N Engl J Med* 1999;341:385–93

6. Garcia PM, Kalish LA, Pitt J, *et al*. Maternal levels of plasma human immunodeficiency virus type 1 RNA and the risk of perinatal transmission. *N Engl J Med* 1999;341:394–402
7. The International Perinatal HIV Group. The mode of delivery and the risk of vertical transmission of human immunodeficiency virus type 1. *N Engl J Med* 1999;340:977–87
8. Perinatal HIV Guidelines Working Group members. Public Health Service task force recommendations for use of antiretroviral drugs in pregnant HIV-1 infected women for maternal health and interventions to reduce perinatal HIV-1 transmission in the United States. Washington DC: US Public Health Service, 2001
9. American College of Obstetricians and Gynecologists. Human immunodeficiency virus infections in pregnancy. ACOG educational bulletin no. 232. Washington, DC: American College of Obstetricians and Gynecologists, 1997
10. American College of Obstetricians and Gynecologists. American Academy of Pediatrics. Joint statement on human immunodeficiency virus screening. ACOG statement of policy. Washington, DC, 1999
11. Stoto MA, Almario DA, McCormick MC, for the Institute of Medicine. *Summary: Reducing the Odds: Preventing Perinatal Transmission of HIV in the United States*. Washington, DC: National Academy Press, 1998
12. American College of Obstetricians and Gynecologists. Scheduled cesarean delivery and the prevention of vertical transmission of HIV infection. ACOG committee opinion no. 234. Washington, DC, 2000
13. Grossman JH, Nies BM, Lopez-Zeno J, *et al*. Management and therapy of human immunodeficiency virus-infected pregnancies in maternal-fetal medicine fellowship training programs. *Obstet Gynecol* 1992;80:985–8
14. Tuomala R, Shapiro D, Samelson R, *et al*. Antepartum antiretroviral therapy and viral load in 464 HIV-infected women in 1998–1999 (PACTG 367). *Am J Obstet Gynecol* 2000;182 (part 2):abstract 285
15. Blanche S, Tardieu M, Rustin P, *et al*. Persistent mitochondrial dysfunction and perinatal exposure to antiretroviral nucleoside analogues. *Lancet* 1999; 354:1084–9
16. Lorenzi P, Spicher VM, Laubereau B, *et al*. Antiretroviral therapies in pregnancy: maternal, fetal and neonatal effects. *AIDS* 1998;12:241

RECEIVED 01/10/01; ACCEPTED 04/23/01

## References

[PDF_00594] Blanche S., Tardieu M., Rustin P., Slama A., Barret B., Firtion G., Ciraru-Vigneron N., Lacroix C., Rouzioux C., Mandelbrot L. (1999). Persistent mitochondrial dysfunction and perinatal exposure to antiretroviral nucleoside analogues.. Lancet.

[PDF_00534] Connor E. M., Sperling R. S., Gelber R., Kiselev P., Scott G., O'Sullivan M. J., VanDyke R., Bey M., Shearer W., Jacobson R. L. (1994). Reduction of maternal-infant transmission of human immunodeficiency virus type 1 with zidovudine treatment. Pediatric AIDS Clinical Trials Group Protocol 076 Study Group.. N Engl J Med.

[PDF_00549] Garcia P. M., Kalish L. A., Pitt J., Minkoff H., Quinn T. C., Burchett S. K., Kornegay J., Jackson B., Moye J., Hanson C. (1999). Maternal levels of plasma human immunodeficiency virus type 1 RNA and the risk of perinatal transmission. Women and Infants Transmission Study Group.. N Engl J Med.

[PDF_00584] Grossman J. H., Nies B. M., Lopez-Zeno J., Bathgate S. L., Parenti D. M. (1992). Management and therapy of human immunodeficiency virus-infected pregnancies in maternal-fetal medicine fellowship training programs.. Obstet Gynecol.

[PDF_00540] Lindegren M. L., Byers R. H., Thomas P., Davis S. F., Caldwell B., Rogers M., Gwinn M., Ward J. W., Fleming P. L. (1999). Trends in perinatal transmission of HIV/AIDS in the United States.. JAMA.

[PDF_00543] Mofenson L. M., Lambert J. S., Stiehm E. R., Bethel J., Meyer W. A., Whitehouse J., Moye J., Reichelderfer P., Harris D. R., Fowler M. G. (1999). Risk factors for perinatal transmission of human immunodeficiency virus type 1 in women treated with zidovudine. Pediatric AIDS Clinical Trials Group Study 185 Team.. N Engl J Med.

[PDF_00531] Rosenberg P. S., Biggar R. J. (1998). Trends in HIV incidence among young adults in the United States.. JAMA.

